# Medical information and decision-making in palliative cancer: a qualitative study of patients’ perceptions, needs, and preferences regarding physician communication

**DOI:** 10.1186/s12904-026-02250-6

**Published:** 2026-08-05

**Authors:** Julia Roick, Christian Heise, Nina Lambrecht, Patrick Michl, Johannes Porzelle, Sophie Rauschenberg, Anke Reinacher-Schick, Henning Rosenau, Jan Schildmann, Theresa Schneider, Karsten Schulmann, Sabine Sommerlatte, Matthias Richter

**Affiliations:** 1 TUM School of Medicine and Health, Department Health and Sport Sciences, Technical University of Munich, Am Olympiacampus 11, Munich, 8080 Germany; 2 Department of Gastroenterology, Hepatology and Infectious Diseases, Heidelberg University Hospital, Heidelberg, Germany; 3 Department of Internal Medicine I, University Hospital Halle, Halle, Germany; 4 Criminal Procedure Law, and Medical Law, Martin Luther University Halle-Wittenberg, Halle, Germany; 5 Hemer Lung Clinic – Center for Pneumology and Thoracic Surgery, Hermer, Germany; 6 Department of Hematology and Oncology with Palliative Care, St. Josef-Hospital, Ruhr- University Bochum, Bochum, Germany; 7 Institute for the History and Ethics of Medicine, Interdisciplinary Center for Health Sciences, Medical Faculty of Martin, Luther University Halle-Wittenberg, Halle, Germany

**Keywords:** Advanced cancer, Physician–patient communication, Medical information, Decision-making, Patient perspectives, Qualitative research methods

## Abstract

**Background:**

Patients with advanced cancer are frequently confronted with complex treatment decisions in the context of high emotional burden and potential limited prognostic benefit. Physician communication and the provision of medical information are therefore central to informed decision-making in the palliative treatment setting. However, little is known about how patients themselves perceive physician communication during oncological informed consent consultations and which needs and preferences they articulate regarding provision of information and decision-making. The aim of this study was to explore patients’ perceptions of physician communication and to identify their needs and preferences in the context of palliative cancer treatment.

**Methods:**

This qualitative study was embedded within a multicenter interventional project in Germany. Semi-structured interviews were conducted with patients diagnosed with stage IV esophageal cancer, stage IV (non-) small cell lung cancer, or Barcelona Clinic Liver Cancer (BCLC) stage C hepatocellular carcinoma who were undergoing systemic therapy. Interviews were conducted either in the outpatient clinic or by telephone, audio-recorded with participants’ consent, and transcribed verbatim. Data were analyzed using qualitative content analysis according to Kuckartz, combining deductive and inductive category development. Coding was performed using MAXQDA.

**Results:**

Analysis identified three central themes. First, communication quality shaped patients’ understanding and coping: patients valued direct, empathic, and transparent communication, but comprehension was often limited by emotional burden, medical terminology, and cognitive overload. Second, treatment decisions were often perceived as predetermined: shared decision-making was limited, and many patients relied on trust-based delegation rather than active decisional control. Third, communication needs were individualized and dynamic: patients differed in their desired information depth and emphasized the need for repeated conversations, plain language, involvement of relatives, and psychosocial support.

**Conclusions:**

Communication in palliative oncology should be conceptualized as an adaptive and ongoing process rather than a single informational event. Patients’ informational needs and preferences for participation vary considerably and are shaped by emotional readiness, prior knowledge, and coping strategies. They should therefore be proactively explored prior to diagnosis and treatment discussions.

**Trial registration:**

The study was registered at the German Clinical Trial Register (DRKS00029632).

**Supplementary Information:**

The online version contains supplementary material available at 10.1186/s12904-026-02250-6.

## Introduction

A cancer diagnosis can lead to severe psychological distress. Approximately one-third of all patients in acute care meet criteria for at least one mental disorder [1–3]. For patients with advanced cancer, prevalence rates are even higher, reaching up to 50% [4, 5]. Symptoms of anxiety and depression as well as uncertainty and loss of control may impair patients’ ability to process complex information and to actively participate in clinical decision-making [6–8]. At the same time, patients with advanced cancer are confronted with complex and far-reaching treatment decisions that often carry profound implications for quality of life, symptom burden, and remaining lifetime. In contrast to earlier stages of the disease, therapeutic options in palliative care are commonly associated with limited prognostic benefit and uncertain outcomes, particularly with respect to survival and quality-of-life outcomes [9, 10]. Treatment decisions therefore often involve weighing potential benefits against substantial burdens, such as treatment-related side effects or reduced physical or psychosocial functioning. Shared decision-making (SDM) provides a conceptual framework for such preference-sensitive decisions by emphasizing a collaborative process in which clinicians and patients exchange information, discuss available options, and integrate clinical evidence with patients’ values, goals, and preferences [11, 12]. SDM has been conceptualized as a collaborative process that differs from both paternalistic decision-making and informed decision-making. Contemporary models further emphasize its iterative and flexible nature, including clear invitations to participate, support in understanding options, elicitation of patients’ goals and preferences, and adaptation to patients’ vulnerability, health literacy, emotional readiness, and desired level of involvement [13–15]. However, treatment decisions frequently occur within time-constrained clinical encounters and under emotionally demanding circumstances, which may limit opportunities for shared decision-making processes [12].

In this context, physician communication during oncological informed consent consultations plays a central role [16]. For patients to provide informed consent and participate in shared decision-making, medical information must be understandable, relevant, and tailored to individual needs. However, achieving this balance is challenging. Excessive or unstructured information may overwhelm patients, while insufficient or overly simplified information may undermine patients’ autonomy and limit their ability to make informed choices. Therefore, physicians face an ethical and clinical challenge to adapt medical information to patients’ decisional capacities, values, and preferences while ensuring transparency and respect for autonomy [17].

Previous research indicates that patients with advanced cancer often have an incomplete or inaccurate understanding of their disease and prognosis. Studies have repeatedly demonstrated discrepancies between physicians’ intentions and patients’ perceptions of the purpose of treatment, with many patients believing that palliative therapies are curative in intent despite limited prognostic benefit [18–20]. Even after interventions to promote understanding (e.g. decision aids, discussions), a substantial proportion of patients continue to hold unrealistic expectations about treatment goals and prognosis [21]. Such misunderstandings may significantly influence treatment preferences and decisions, potentially leading to care that is misaligned with patients’ values or goals. While quantitative studies have provided important insights into the prevalence and consequences of limited prognostic understanding, less is known about how patients themselves experience physician communication and the process of receiving medical information in palliative care. In particular, there is a lack of in-depth qualitative research exploring how patients emotionally and cognitively process prognostic and treatment-related information over time, and how communication may become either manageable or overwhelming in palliative care contexts. Understanding these perspectives is essential for developing patient-centered interventions that are responsive to patients’ vulnerabilities, informational needs, and ethical concerns.

The aim of this qualitative study was to explore how patients under palliative cancer treatment perceive physician communication regarding medical information and decision-making during informed consent consultation. In particular, the study sought to examine how emotional distress, timing, language, and relational aspects of communication shape whether medical information becomes understandable, emotionally manageable, and meaningful for patients in palliative care contexts.

## Patients and methods

### Study design

This exploratory qualitative study was embedded within a larger, multicenter interventional study aiming to develop and pilot a multimodal intervention to improve decision-making capacity among patients with advanced cancer. Inclusion criteria were (1) 18 years and older, (2) diagnosed cancer of the esophagus stage IV or (non-) small cell lung carcinoma stage IV or Barcelona Clinic Liver Cancer (BCLC) stage C hepatocellular carcinoma, (3) indication for a systemic therapy, and (4) sufficient German language skills to participate in an interview. Patients in the dying phase were excluded. The qualitative component was designed to explore patients’ experiences, needs, and preferences regarding physician communication and medical information in the context of advanced cancer care. The qualitative component aimed to generate patient-centered insights relevant to the development of the intervention materials. This study was granted ethical approval by the Ethics Committee of the Medical Faculty at Martin Luther University Halle-Wittenberg (#2023-30) and registered at the German Clinical Trial Register (DRKS00029632; https://www.drks.de) on May 30, 2023.

### Data collection

Participants were recruited using consecutive purposive sampling based on the predefined eligibility criteria among three hospitals in Germany (Halle [Saale], Heidelberg, Bochum). Data were collected by two trained researchers (JR PhD, TS M.mel.) who were part of the study team. They conducted semi-structured interviews (see supplement A) either in the clinic or via telephone when the patient had returned home. Both interviewers were female and had received formal training in qualitative research methods. JR has background in sociology and psychology, and TS in medicine, ethics, and law. The researchers were not involved in the clinical care of the participants and had no prior relationship with them. Eligible participants were identified and approached by the clinical staff who informed them about the aim and scope of the study. After providing written informed consent, patients were contacted by the researcher to schedule a time and place for the interview. No relatives or clinical staff were present during the interviews.

The interview guide is based on the study’s research questions and was further informed by existing literature on prognostic awareness [19–21], shared decision-making [12, 22], and communication in palliative oncology [17, 23, 24]. Previous research highlighting patients’ difficulties in understanding prognosis and treatment goals, emotional barriers to information processing, and variability in preferences for participation in decision-making informed the selection of predefined interview topics. The interview guide was designed to explore both informational and relational dimensions of communication in serious illness contexts. The final version consisted of open-ended questions addressing predefined topics in order to obtain an in-depth understanding of the areas of interest. Predefined interview domains included patients’ perceptions of the process of receiving medical information, participation in decision-making, understanding of prognostic and treatment-related information, and suggestions for improving physician communication and patient information. Participants were also encouraged to introduce additional issues they considered relevant to their experiences and perspectives. In addition, a brief standardized questionnaire was administered during the interview to collect demographic information. Field notes were taken after each interview to document contextual information. The interview guide was pilot tested prior to the start of patient recruitment and refined accordingly. With participants’ permission, all interviews were digitally audio-recorded and subsequently transcribed verbatim by an external professional transcription service. No repeat interviews were conducted, and transcripts were not returned to participants for comment. Interviews were conducted until thematic saturation was reached, understood as the point at which no substantially new themes or subthemes emerged during ongoing analysis [25].

### Data analysis

Data were analyzed using qualitative content analysis following the approach described by Kuckartz [26]. The analysis combined deductive and inductive category development in a systematic and iterative process. An initial coding framework was derived from the research questions and the interview guide. During close reading of the transcripts, additional categories and subcategories were developed inductively to capture emerging themes. Coding was performed using the qualitative data analysis software MAXQDA. Coding was conducted by JR and discussed regularly within the research team. The research team included members with backgrounds in sociology, psychology, medicine, ethics, and medical law. To enhance analytical rigor, 30% of the transcripts were independently coded by TS. Discrepancies were resolved through discussion until consensus was reached. Results are reported following the consolidated criteria for reporting qualitative research (COREQ) [27].

## Results

### Sample characteristics

Overall, 23 interviews were conducted and lasted approximately 8–51 min. Characteristics of the sample can be seen in Table 1.

**Table 1:** Sample characteristics

Category	Value (*n* = 23)
Age in years, mean (Min.; Max.)	65.3 (43; 75)
Gender (%)	
Female	43.5
Male	56.5
Education (%)	
Vocational training	91.7
Higher education	8.3
Primary Tumor site (%)	
Lung	82.6
Liver	8.7
Esophagus	8.7

### Overview of thematic findings

**Fig. 1:**
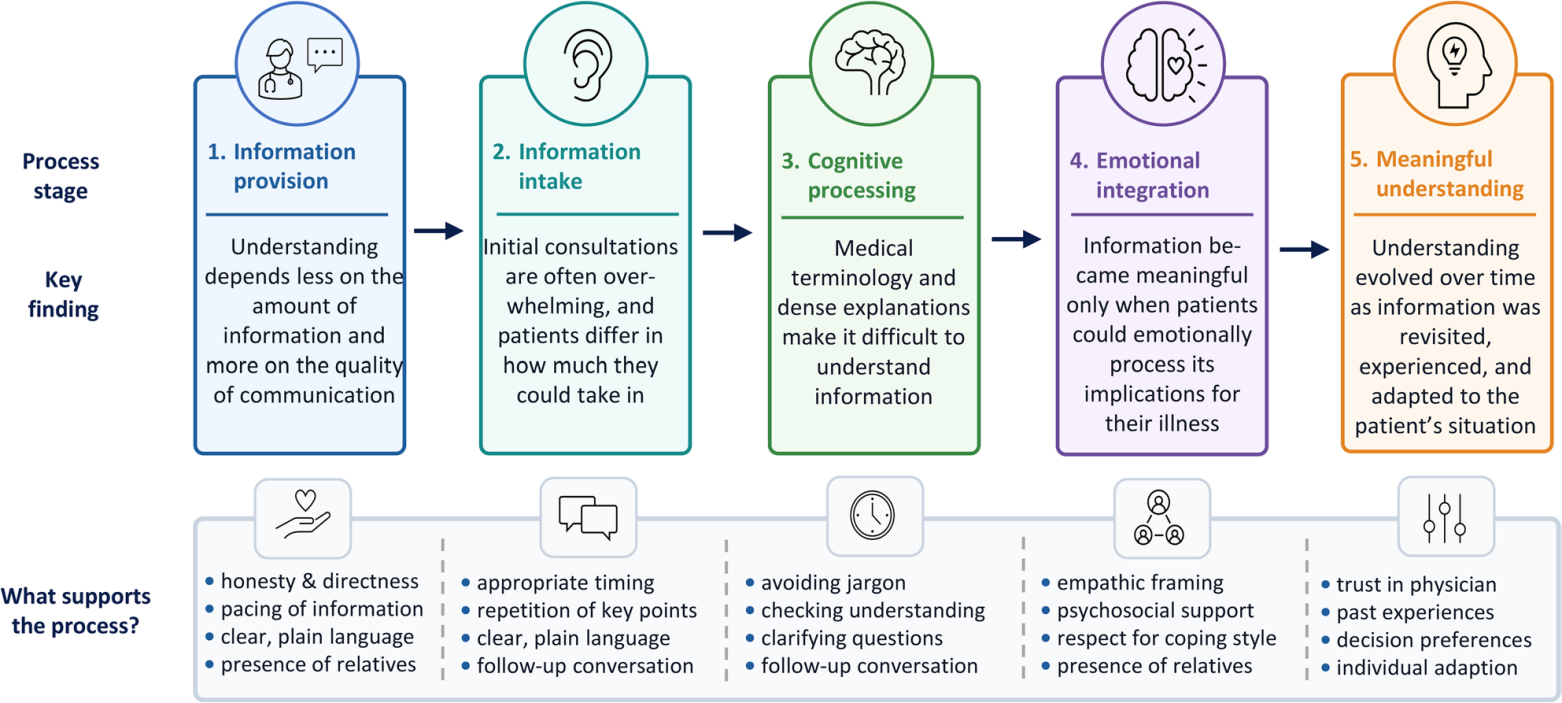
Conceptual synthesis of communication processes in palliative oncology

Analysis of the patient interviews showed that patients did not primarily evaluate communication according to the amount of information provided, but according to whether information was delivered in a way that was emotionally manageable, individually adaptable, and cognitively processable. Across interviews, patients described communication as a dynamic process in which different factors shaped whether information could be meaningfully understood and integrated into decision-making. The analysis generated three overarching themes, each comprising several subthemes that reflect different dimensions of patients’ experiences and communication needs. Figure 1 provides a conceptual synthesis of the thematic findings and illustrates the process described across patients, from information provision to information intake, cognitive processing, emotional integration, and meaningful understanding.

### Communication quality influences patients’ understanding and coping

Patients described physician communication as supportive when physicians combined clear explanations with empathy, sufficient time, and responsiveness to emotional needs. Across interviews, participants emphasized that their ability to understand and cope with their illness depended less on the amount of information provided and more on how communication was relationally delivered and emotionally framed. Communication was experienced positively when physicians responded sensitively to emotional needs and created opportunities for clarification and dialogue.

#### Empathy and directness shaped communication experiences

Participants associated high-quality communication primarily with relational and emotional aspects of physician–patient interactions. Positive experiences were characterized by openness, sufficient time, and a respectful attitude toward patients’ questions and concerns. Even difficult information, such as the palliative nature of treatment, was described as acceptable when delivered transparently and with sensitivity. As one participant reflected:

*“She was very direct and didn’t sugarcoat anything. But she was also understanding – so that even I understood it.” (P5)*.

Patients repeatedly emphasized the importance of not feeling rushed and of being taken seriously. Communication was experienced as high quality when physicians signaled availability beyond a single encounter and created space for clarification and dialogue. In these accounts, communication was understood as an ongoing relational process rather than a one-time transfer of medical facts.

*“Those were the questions I had. And all the other questions I had before and after were answered before and after*,* in detail and with patience and personal empathy.” (P12)*.

In contrast, negative experiences were associated with perceived gaps in information, emotional distance, or a lack of continuity. Some participants described interactions as fragmented or impersonal, reporting that they felt insufficiently informed despite having formally consented to treatment. Others referred to a mismatch in interpersonal dynamics, where communication felt strained or unresponsive.

*“The chemistry with the doctors didn’t fit. No communication. You had to beg for everything.” (P6)*.

Importantly, dissatisfaction was rarely attributed to the content of information alone. Rather, it was the tone and relational context of communication that shaped patients’ evaluations. Overall, perceived quality appeared to depend less on whether the message was positive or negative and more on how relationally embedded and responsive the interaction was.

#### Emotional distress limited comprehension of medical information

Although most participants reported that physicians provided detailed explanations regarding diagnosis and treatment, actual comprehension was often limited by emotional distress, cognitive overload, and complex medical terminology. Some patients described feeling well informed and able to follow the structure of their therapy, including treatment cycles and monitoring procedures.

*“He showed me how it’s going to happen now*,* these four blocks*,* right? The chemo there*,* that you do that and then in the meantime CT scans are done again.” (P12)*.

However, other patients revealed substantial gaps in understanding. In extreme cases, they reported not knowing the exact type of cancer they had.

*“Well*,* what can I say? I didn’t even know what kind of cancer I had. I had no idea. I didn’t know that at all.” (P1)*.

Medical terminology emerged as a recurring barrier. Several participants indicated that explanations contained unfamiliar terms that required independent clarification.

*“For some words*,* we had to look them up on the internet to see what they actually meant.” (P5)*.

Even when explanations were thorough, the density and complexity of information could exceed patients’ cognitive capacity, particularly in emotionally challenging situations. One participant described how extended explanations made it difficult to formulate follow-up questions.

*“When a doctor explains something for five minutes and there are so many things in it that you didn’t really understand*,* you can’t even ask about everything*,* because you can’t repeat it.” (P13)*.

Beyond immediate comprehension, participants highlighted a distinction between cognitive understanding and experiential comprehension. Information that seemed understandable during the consultation could take on a different meaning once patients encountered the lived reality of treatment.

*“But there is still a world of difference between explaining*,* understanding*,* and experiencing. That’s just how it is.” (P9)*.

Across interviews, participants described three recurring barriers to understanding: emotional burden during consultations, complex medical terminology, and the difficulty of relating information to the lived reality of treatment. Several patients indicated that information could be heard during the consultation but only become understandable or personally meaningful later.

### Treatment decisions were often perceived as predetermined

Many participants described treatment decisions as largely determined within institutional and medical structures rather than as actively negotiated choices. While a few patients reported feeling informed and involved, most described decision-making processes as primarily structured. Participation was therefore often perceived as limited, even when formal consent was obtained. Explicit experiences of shared decision-making were rare. Several participants stated that treatment options were not actively discussed as choices:

*“[…] the question [about treatment choice] was not even asked.” (P3)*.

In some cases, treatment pathways were presented as predefined. In such situations, decisions were understood as having been determined within institutional processes. Here, trust in the institutional and medical framework replaced active decision-making. The decision was perceived as medically established rather than jointly negotiated. One participant stated.

*“So*,* there’s this tumor board meeting every Thursday at the clinic. And that’s where the case was discussed. And I assumed that they had already chosen the best option.“. (P7)*

Even when formal autonomy was acknowledged, it was often experienced as limited in practice. Some experienced a gap between theoretical choice and perceived feasibility. So, although refusal was practically possible, it was not experienced as a realistic option.

*“Of course*,* I could have said*,* “No*,* I don’t want to do that*,*” but I don’t think that would have been a good decision.” (P2)*.

#### Trust-based delegation reduced decisional burden

Preferences regarding involvement in decision-making differed among participants, but explicit demands for active participation were rare. Most patients did not describe a strong wish to influence treatment choices directly. Instead, many patients reflected acceptance of medically proposed treatment plans. Trust in medical expertise was often cited as the reason for this preference.

*“Let me put it this way*,* most people think*,* “What the doctor says must be right.” (P13)*.

This statement reflects a broader orientation toward medical authority that shaped participation beyond individual encounters. Further, being asked to make complex treatment decisions in a palliative context could feel burdensome rather than empowering. Delegation was therefore not necessarily a sign of passivity, but an expression of reliance on professional judgment. Also, distancing from decision-making could function as a coping strategy in the face of existential threat.

*“So*,* what I don’t know doesn’t bother me*,* right?” (P7)*.

In the interviews, delegation was linked to trust in medical expertise, perceived lack of realistic alternatives, and the emotional burden of making complex treatment decisions.

### Patients preferred individualized and adaptive communication

Participants emphasized that effective communication in palliative care requires adaptation to individual emotional, cognitive, and situational needs. Rather than preferring standardized information delivery, patients described communication as most helpful when physicians adjusted timing, pacing, language, and emotional framing to the patient’s current condition and coping capacity. Informational needs were experienced as dynamic and subject to change over time.

#### Patients needed repeated conversations over time

Patients emphasized that the timing and pacing of information were crucial. Several described the initial consultation as emotionally overwhelming, particularly when extensive prognostic and therapeutic information was delivered at once. Even when physicians provided comprehensive explanations, patients reported that they were unable to absorb the information at that time.

*“Because I just got the diagnosis and then it turned off for me. […] They definitely told me that*,* but I didn’t take it in that day.” (P14)*.

This description reflects a state of cognitive shutdown in response to emotional shock. Although information may have been provided, it was not cognitively retained. Patients described this as a state in which further details could not be absorbed, regardless of the clarity of explanation. As a consequence, participants highlighted the importance of temporal structuring in communication and expressed the need for follow-up conversations.

*“So normally there would have to be a second consultation*,* because as I said*,* you can’t take all that in anymore.” (P13)*.

The wish for subsequent discussions was not framed as dissatisfaction with the physician, but as recognition that understanding evolves over time. Patients indicated that being able to revisit topics and clarify uncertainties at a later stage would support comprehension and decision-making.

#### Preferences for prognostic information varied

Participants expressed heterogeneous preferences regarding the amount of information they wished to receive. While some desired comprehensive explanations, others explicitly preferred limited detail as a strategy of emotional self-protection.

*“But that wasn’t what I wanted personally. I didn’t want to delve too deeply into the subject matter.” (P3)*.

For these individuals, restricting information functioned as a coping mechanism. Excessive detail was described as potentially increasing psychological distress. At the same time, some participants articulated conditions under which they would continue treatment, linking medical information to personal thresholds of self-determination. One participant noted that continuing therapy depended on clear prognostic communication and a realistic assessment of benefit.

*“But if I don’t get any information on what the chances are*,* if there’s an 80% chance that it will actually work out*,* so I say okay*,* I might continue [with chemotherapy]*,* but under that*,* you can forget it.” (P9)*.

This illustrates that patients differed in how much prognostic detail they wished to receive and how closely they wanted information to be linked to treatment decisions.

#### Communication needs depended on prior knowledge and coping style

A central theme was the need for communication to be adapted to individual circumstances, capacities, and preferences. Participants emphasized that physicians should actively explore how much information a patient wishes to receive and adjust explanations accordingly. Differences in prior knowledge played a role in shaping informational needs.

*“I worked for a pulmonologist for 20 years […] so there was no need to explain everything to me in detail.” (P19)*.

Here, medical familiarity reduced the need for extensive elaboration. For some participants with professional medical backgrounds, informational needs differed compared to those with no medical background. In contrast, other participants struggled with medical terminology and expressed a desire for clearer, less technical language.

*“They talk and talk and I can hardly understand anything*,* so I understand almost nothing. What they mean and all that*,* I don’t know anything about that. […] If they would at least say it clearly or say it in German and not here*,* what do I know*,* Latin. I don’t know Latin.” (P1)*.

These contrasting accounts illustrate that informational adequacy cannot be assessed independently of the recipient’s background and comprehension capacity. Participants also highlighted contextual aspects of communication. Some expressed a wish for more structured involvement of relatives, particularly when facing complex treatment decisions. Others referred to a general need for more accessible psychological support in connection with emotionally demanding communication, but did not consistently specify whether this support should be provided during the consultation itself or afterwards.

*“Psychological support could be more comprehensive.” (P7)*.

Overall, it appears that communication needs varied according to prior knowledge, comprehension capacity, emotional state, preferred information depth, and the availability of social or psychological support.

## Discussion

This study explored how patients receiving palliative cancer treatment perceive physician communication and decision-making processes during oncological informed consent consultations. The findings suggest that the central challenge of communication in palliative oncology is not merely the provision of accurate medical information, but the ability to adapt communication to patients’ emotional readiness, cognitive processing capacity, coping style, and situational needs. Patients described meaningful understanding as a dynamic and relational process rather than a simple transfer of information. The study therefore extends existing research on prognostic communication by showing that informational adequacy depends not only on what is communicated, but also on how, when, and under which emotional conditions information is delivered and revisited.

### Quality and understanding in communication

Across interviews, the perceived quality of communication was shaped less by the amount of information provided and more by the relational context in which it was delivered. Patients emphasized empathy, sufficient time, and responsiveness as determining elements. Even difficult information, such as the palliative intent of treatment, was described as acceptable when communicated transparently and sensitively. This finding aligns with previous research showing that patients value compassionate and individualized communication in advanced cancer care [23, 24]. It also resonates with indications that oncologists are often reluctant to discuss prognosis directly and may not always recognize signals from patients that indicate their willingness to engage in more explicit communication, or may generally fail to respond to such signals [28]. Our findings suggest that the manner and relational framing of information may be more consequential than the factual content alone.

Despite reports of detailed explanations, many participants described substantial gaps in understanding. Medical language and cognitive overload, particularly at the moment of diagnosis, limited retention and meaningful processing. Patients’ perceptions illustrate that comprehension is not a static achievement, but an evolving process shaped by emotional readiness and lived experience. These observations are consistent with other studies demonstrating that patients with advanced cancer frequently have incomplete or inaccurate understanding of prognosis and treatment goals [29, 30]. A systematic review also indicates that physicians often face barriers in prognostic and palliative care discussions, leading to reactive rather than proactive communication patterns [24]. Importantly, our findings extend this literature by highlighting that patients distinguished between hearing medical information, cognitively understanding it, and emotionally integrating its implications into their lived reality. This suggests that informational adequacy cannot be assessed solely by whether facts were communicated or not.

### Participation in decision-making

Shared decision-making was limited in many patients. Treatment pathways were often perceived as institutionally predetermined, with options framed as medically necessary rather than negotiable. Although formal autonomy existed in principle, patients frequently experienced refusal as unrealistic. These findings complicate normative models of shared decision-making. Rather than striving uniformly for active participation, many patients preferred trust-based delegation. Trust in professional expertise and tumor board decisions often substituted for direct involvement. However, the preference for trust-based delegation should not be interpreted as evidence that patients would not benefit from better supported shared decision-making. Some patients may have delegated decisions because treatment options were presented as medically predetermined or because the relevance of their values and preferences for treatment decisions was not made sufficiently explicit. Future communication strategies should therefore not simply ask whether patients want to participate, but should explain why their preferences may matter and provide structured support for involvement, while also respecting informed delegation when this remains the patient’s preference. In some cases, distancing from decision-making appeared to function as an emotional coping strategy. Participation in decision-making in this context therefore appeared to be shaped by an interplay of perceived choice, trust, and emotional coping, rather than by the availability of treatment options alone. This suggests that participation in palliative contexts may not primarily reflect deficits in autonomy but rather context-sensitive forms of relational autonomy. Patients’ preferences varied, and active involvement was not universally desired. Similar patterns have been observed in studies indicating that patients’ desire for decision-making involvement fluctuates depending on disease stage, emotional burden, and socio-economic factors like age or education [22, 31, 32].

### Individual needs and preferences

A key contribution of this study lies in the emphasis on individual needs and preferences. One point is the tension between honesty and emotional protection as an enduring challenge in oncology communication [33]. Our results show that patients did not uniformly demand maximal transparency. Rather, patients sought communication that balanced honesty with sensitivity to emotional limits. Informational needs were closely intertwined with personal values, coping strategies, and notions of self-determined decision-making. Other studies also conclude that there are varying preferences regarding diagnostic information, with less information being desired particularly in cases with poorer prognoses [34, 35]. Informational needs were shaped by patients’ prior medical knowledge and individual coping styles. While participants with greater medical knowledge often felt less need for detailed explanations, some patients employing avoidant coping strategies preferred to limit the amount of information they received in order to avoid emotional distress. This suggests that effective communication requires flexible approaches rather than standardized formats. In addition, participants emphasized the importance of structured involvement of relatives in medical discussions, underscoring findings that family inclusion plays a critical role in cancer care [36]. The expressed need for enhanced psychological support during emotionally demanding conversations further highlights that communication needs in palliative oncology extend beyond the transmission of medical content and include support for emotional processing and coping.

Taken together, our findings suggest that effective communication in palliative care requires flexible adaptation to individual informational needs, emotional states, cognitive capacities, and social contexts. Rather than relying on a standardized communication model, clinicians may need to provide situationally responsive and personalized dialogue.

### Strengths and limitations

Several limitations should be considered when interpreting the findings. First, selection bias cannot be excluded. Patients experiencing particularly high emotional distress or psychological burden may have been less likely to participate in an interview. As a result, perspectives of those with potentially greater communication needs or different informational preferences may not be fully represented in the present sample. Second, some interviews were relatively short, limiting the depth of exploration in certain cases. This may indicate restricted emotional capacity or limited readiness to elaborate on sensitive topics. Consequently, some nuances of communication experiences may not have been fully captured. Third, the data are based on retrospective self-reports and therefore reflect subjective interpretations of past encounters. Recall bias and emotional framing may have influenced how experiences were narrated. Additionally, interviews conducted within clinical environments may have been shaped by contextual factors such as ongoing treatment relationships. Despite these limitations, the study has several strengths. Interviews were conducted in real-world palliative care settings, allowing patients to articulate their perspectives within the context in which communication actually occurred. The focus on patients with advanced cancer provides insights into communication processes at a particularly vulnerable stage of illness. By exploring not only informational aspects but also relational and contextual dimensions, the study contributes nuanced understanding to the literature on communication in palliative oncology.

### Practical implications

Table 2 summarizes how the findings extend existing knowledge on communication in oncology and outlines potential implications derived from the results.

**Table 2:** Comparative synthesis of known evidence, study findings, and implications

What is known	What we found in this context	Potential implications from the results
Empathic communication is valued in oncology.	Patients accepted even difficult prognostic information when delivered relationally and without time pressure	Communication training should focus on relational delivery, not only content.
Patients with advanced cancer often misunderstand prognosis and treatment goals.	Patients distinguished between hearing information and emotionally processing it.	Understanding should be checked. Follow-up consultations and inclusion of relatives may be necessary for meaningful comprehension.
Shared decision-making is promoted in oncology.	Many patients preferred trust-based delegation.	Clinicians should assess desired level of participation individually.
Information preferences vary between patients.	Some patients intentionally limited information as emotional self-protection. Needs also depend on coping style and prior knowledge.	Communication should be individualized according to patients’ coping style, emotional readiness, and health literacy.

Overall, the results suggest that communication in palliative oncology should be understood as an adaptive and relational process rather than a single transfer of medical information. First, clinicians should recognize that patients’ understanding depends not only on the amount of information provided, but also on how information is delivered. Direct and honest communication was appreciated when it was embedded in an empathic and respectful interaction. Communication training should therefore address relational delivery, emotional responsiveness, and the ability to communicate difficult prognostic information clearly without increasing distress. Second, information should be revisited over time.

Initial informed consent conversations may exceed patients’ cognitive processing capacity, particularly in emotionally overwhelming situations. Proactively scheduling follow-up consultations and explicitly inviting patients to revisit questions may support gradual comprehension over time. Third, in palliative care settings, patients’ preferences regarding the depth of information and their desired level of involvement in decision-making should not be assumed, but explicitly explored. Rather than uniformly promoting shared decision-making, clinicians should assess whether patients prefer active participation or trust-based delegation. Attention should be paid to linguistic clarity and avoidance of unnecessary medical terminology. Even when explanations appear straightforward from a clinical perspective, comprehension cannot be presumed. Interactive strategies, such as encouraging patients to paraphrase information or inviting clarifying questions, may help ensure mutual understanding. Finally, communication should be individualized according to patients’ coping style, emotional readiness, prior knowledge, and health literacy. Some patients wanted detailed information, whereas others intentionally limited information to protect themselves emotionally. Particularly in palliative contexts, communication should be embedded within a broader framework of relational and psychosocial care.

## Supplementary Information

Supplementary Material 1.

Supplementary Material 2.

## Data Availability

The datasets generated and/or analyzed during the current study are not publicly available due to the sensitive and potentially identifiable nature of qualitative interview data. However, anonymized excerpts may be available from the corresponding author on reasonable request.
