# Supplementary material for: Medical information and decision-making in palliative cancer: a qualitative study of patients’ perceptions, needs, and preferences regarding physician communication
Source: BMC Palliat Care. 2026 Aug 5;25:227. doi: 10.1186/s12904-026-02250-6 (PMC13440109; doi:10.1186/s12904-026-02250-6)
Supplement: Supplementary file 1 [file 12904_2026_2250_MOESM2_ESM.pdf]

Supplement B

Coding scheme to structure the qualitative findings

Main theme	Subtheme	Code definition	Exemplary quote
Perception of the informed consent consultation	Relational aspects of communication	Segments in which participants described the interpersonal quality of physician–patient communication, including feeling respected, taken seriously, listened to, or able to ask questions.	Those were the questions I had. And all the other questions I had before and after were answered before and after, in detail and with patience and personal empathy. (P12)
	Emotional aspects of communication	Statements referring to the emotional tone of communication, including empathy, sensitivity, emotional responsiveness, or the way difficult information was delivered.	She was very direct and didn't sugarcoat anything. But she was also understanding – so that even I understood it. (P5)
	Cognitive aspects of information processing	Segments in which participants described difficulties absorbing, retaining, or following information during consultations because the information was too dense, complex, lengthy, or emotionally overwhelming.	When a doctor explains something for five minutes and there are so many things in it that you didn't really understand, you can't even ask about everything, because you can't repeat it. (P13)
	Structural aspects of the consultation	Statements referring to the organization and structure of consultations, including available time, pacing, opportunities for clarification, or the possibility of follow-up conversations.	Those were the questions I had. And all the other questions I had before and after were answered before and after, in detail and with patience and personal empathy. (P12)
Illness- and treatment-related understanding	Understanding of treatment structure	Segments in which participants described understanding the planned treatment process, including treatment steps, cycles, monitoring procedures, or the sequence of care.	He showed me how it's going to happen now, these four blocks, right? The chemo there, that you do that and then in the meantime CT scans are done again. (P12)
	Substantial gaps in illness understanding	Statements in which participants described limited or absent understanding of basic illness-related information, such as cancer type, disease status, treatment intent, or prognosis.	Well, what can I say? I didn't even know what kind of cancer I had. I had no idea. I didn't know that at all. (P1)
	Medical terminology as barrier	Segments in which participants referred to difficulties understanding medical terminology, technical language, abbreviations, or unfamiliar clinical terms.	For some words, we had to look them up on the internet to see what they actually meant. (P5)
	Experiential understanding	Statements in which participants distinguished between receiving or cognitively understanding information and fully grasping its meaning through lived experience with illness or treatment.	But there is still a world of difference between explaining, understanding, and experiencing. That's just how it is. (P9)

Main theme	Subtheme	Code definition	Exemplary quote
Decision-making regarding treatment	Perceived absence of real treatment choice	Segments in which participants described treatment decisions as medically or institutionally predetermined, or in which formal options such as refusing treatment were not experienced as realistically feasible.	[…] the question [about treatment choice] was not even asked. (P3)
	Trust-based delegation and emotional coping	Segments in which participants described relying on physicians' expertise, accepting medical recommendations, or preferring to delegate treatment decisions to the medical team to reduce emotional burden.	What the doctor says must be right. (P13)
	Information linked to personal treatment thresholds	Segments in which participants described using prognostic or treatment-related information to evaluate whether continuing therapy would meet their personal thresholds for acceptable benefit, chance of success, or treatment burden.	If I don't get any information on what the chances are, if there's an 80 percent chance that it will actually work out, so I say okay, I might continue [with chemotherapy], but under that, you can forget it. (P9)
Communication needs and preferences	Need for repeated conversations	Statements indicating a need to revisit information or have follow-up consultations after initial diagnostic or treatment discussions.	So normally there would have to be a second consultation, because as I said, you can't take all that in anymore. (P13)
	Need for plain language	Statements referring to a desire for simpler, clearer, less technical, or more everyday language in explanations.	If they would at least say it clearly or say it in German and not here, what do I know, Latin. I don't know Latin. (P1)
	Involvement of relatives	Segments referring to the presence, inclusion, or desired involvement of relatives in communication, information processing, or decision-making.	And that's why it's so helpful for me, really helpful, to go through the brochures together [with the wife] or to have her come along to these consultations. (P5)
	Psychosocial support needs	Statements referring to the need for psychological, psychosocial, or emotional support during or after diagnosis disclosure.	Psychological support could be more comprehensive. (P7)
	Preferred information depth	Segments in which participants described how much information they wanted to receive, including preferences for more detailed information, selective information, or limited information.	But that wasn't what I wanted personally. I didn't want to delve too deeply into the subject matter. (P3)
	Prior medical knowledge	Segments in which participants described prior medical knowledge, professional experience, or familiarity with healthcare as influencing the amount, level of detail, or type of information they needed.	I worked for a pulmonologist for 20 years [...] so there was no need to explain everything to me in detail. (P19)
