# Supplementary material for: Medical information and decision-making in palliative cancer: a qualitative study of patients’ perceptions, needs, and preferences regarding physician communication
Source: BMC Palliat Care. 2026 Aug 5;25:227. doi: 10.1186/s12904-026-02250-6 (PMC13440109; doi:10.1186/s12904-026-02250-6)
Supplement: Supplementary file 2 [file 12904_2026_2250_MOESM1_ESM.docx]

Supplement A

**Interview guide: Communication in palliative cancer**

| **Key question** | **Themes** |
| --- | --- |
| At the beginning of the interview, I would like to ask you to tell me your cancer history. | - Entity - Course of treatment - Current state of treatment |
| Next, I would like to ask you to tell us about the process of receiving information about your cancer from your doctor.  How did you feel about the information process? | - Course of the communication process about the diagnosis - Did patients felt well informed, did they ask questions - Identification of possible shortcomings in the communication about diagnosis and treatment - Personal feelings about positive and negative characteristics during conversation |
| How was the treatment decided? | - Were various options proposed? - Who made the decision for the current course of treatment? |
| How did you perceive difficulties regarding the explanation of your cancer diagnosis and treatment? | - Difficulties in communication - Negative effect on the patient |
| Do you have any suggestions for solutions or improvements to the difficulties you experienced during the conversation? | - What are the wishes and needs for future conversations? |
| At the end of the interview, I would like to ask you if there is anything on this topic in general that is important from your point of view. | |
